# Interfacial passivation of CsPbI_3_ quantum dots improves the performance of hole-transport-layer-free perovskite photodetectors

**DOI:** 10.1186/s11671-023-03793-w

**Published:** 2023-02-13

**Authors:** Houpu Zhou, Mengwei Chen, Chenguang Liu, Rui Zhang, Jing Li, Sainan Liao, Haifei Lu, Yingping Yang

**Affiliations:** 1 Department of Physics, School of Science, Wuhan University of Technology, Wuhan, China; 2 Hubei Engineering Research Center of RF-Microwave Technology and Application, Wuhan University of Technology, Wuhan, 430070 China

**Keywords:** Perovskite photodetectors, CsPbI_3_ quantum dot, Interface passivation, Energy level matching

## Abstract

Photodetectors (PDs) suffer from dark current due to defects in the perovskite photosensitive layer. Contact between the photosensitive layer and carbon electrodes could result in recombination of carriers at the interface. In this work, CsPbI_3_ quantum dots (QDs) were added between the photosensitive layer and the carbon electrode as the interfacial layer to passivate the surface defects of perovskite layer and improve the energy level matching at the interface. The effect of QDs concentrations on the passivation of the perovskite layer was investigated. It was found that the photoluminescence intensity of perovskite films was the strongest and the decay lifetime was the longest when the QDs concentration was 3 mg/mL. Owing to QDs passivation, the dark current of perovskite PD decreased by 94% from $2.04\; \times \;10^{ - 9}$ to $1.17\; \times \;10^{ - 10}$ A. The responsivity (*R*) at 605 nm improved by 27% from 0.29 to 0.37 A/W at 0 V bias voltage. The specific detectivity (D*) increased by 420% from $8.9\; \times \;10^{11}$ to $4.7\; \times \;10^{12}$ Jones.

## Introduction

Photodetectors (PDs) can convert light signals into electrical signals. They are an imperative component of optical communication, sensing technology, electronic imaging, and other fields [1]. Since the first photoelectric device based on perovskite semiconductor material was successfully prepared in 2009, perovskite semiconductor materials have been rapidly developing for photovoltaic, luminescence and photoelectric detection applications [2]. In addition to possessing excellent optical and electrical properties, perovskites are typically made through inexpensive raw materials and synthesis [3]. Its advantages make it a promising semiconductor material. At present, the responsivity (*R*) of PDs based on organic perovskite material CH_3_NH_3_PbI_3_ (MAPbI_3_) is close to that of commercial silicon PDs. The specific detectivity (D*) in the same band is even one order of magnitude higher than that of silicon PDs, and the response time reaches nanosecond level [4, 5].

In order to achieve PDs with higher *R* and higher D*, it is necessary to reduce the dark current of the devices. The dark current of PDs mainly comes from internal and surface defects in the crystal film [6]. The dark current of PDs is primarily caused by structure defects and charge trapping in the photosensitive layer, which constitutes the most significant portion of the detector. The defect in the photosensitive film also lead to increased charge carrier recombination, which seriously affects the photoelectric performance of PDs [7]. Currently, there are many studies reducing the dark current of devices by optimizing the perovskite photosensitive layer. For example, after the electron transport layer is treated with tetramethylammonium hydroxide (TMAOH) and polyaniline (PANI), the perovskite film deposited on it has higher quality and a better spreading effect [8, 9]. In addition, bathocuproine (BCP) and fullerene (C_60_) as the interface layer in the electron transport layer can passivate the rough surface of the perovskite photosensitive layer and reduce the injection of holes [10]. Doping other substances such as CdCl_2_, quantum dots (QDs) and nitrosonium tetrafluoroborate (NOBF_4_) in the precursor solution of the photosensitive layer could promote the growth of perovskite films [11–15]. Addition of KBr or QDs to the antisolvent can also increase the crystallinity of perovskite films [16].

Although significant progress has been made to improve the performance of perovskite PDs, the current high-performance devices are based on expensive hole transport materials, such as 2,2′,7,7′-tetrakis(*N*,*N*′-di-p-methoxyphenylamine)-9,9′-spirobifluorene (Spiro-OMeTAD) and poly[bis(4-phenyl)(2,4,6-trimethylphenyl) amine] (PTAA). Adding a multilayer barrier layer to the carrier transport layer can reduce dark current of devices, which significantly increases the process complexity and costs associated with device preparation. This is not conducive to the commercialization of PDs. Because perovskite materials themselves have the characteristics of bipolar carrier transport, the construction of PDs with a simple structure can be realized by eliminating the hole transport layer [17]. However, the PD with hole-transport-layer-free still has many problems, among which the most obvious one is the large charge carrier recombination at the interface due to the direct contact between the electrode and the photosensitive layer. Therefore, further investigation of hole-transport-layer-free PDs is needed.

In recent years, all-inorganic perovskite QDs have attracted significant attention in various fields due to their excellent photoelectric properties and quantum effects [18]. In the field of solar cells, perovskite QDs have been successfully applied to the light absorption layer, interface modification layer and carrier transport layer [19–21]. Furthermore, in the past few years, many works on PDs based on perovskite QDs photosensitive layers such as CsPbBr_3_ QDs and CsPbI_3_ QDs have been reported [22, 23]. Successful synthesis of perovskite QD_S_ under open atmospheric conditions has also been reported [24]. However, the combination of perovskite QDs with perovskite PDs is rarely studied. Perovskite QDs have similar lattice constants to those of perovskite photosensitive layers [25] and are excellent materials for interfacial passivation. Thus, the surface of the photosensitive layer can be passivated using perovskite QDs through interface engineering, which can not only reduce the surface defects, but also increase the energy level matching at the interface by the band structure of perovskite QDs [26].

Based on the structure of hole-transport-layer-free perovskite PD with carbon electrode, we studied the concentration effect of CsPbI_3_ QDs on the passivation of perovskite interface and obtained the perovskite photosensitive layer with low surface defects. When the concentration of CsPbI_3_ QDs was 3 mg/mL, the defect passivation effect of perovskite films was the best with the strongest PL intensity and longest decay lifetime of the films. After QDs passivation, the dark current of the perovskite PD decreased from $2.04 \times 10^{ - 9}$ to $1.17 \times 10^{ - 10}$ A, and D* of the device increased significantly.

## Methods

### Materials

Oleic acid (OA, 99%), oleylamine (OAM, 80–90%), 1-octadecene (ODE, > 90%), methyl acetate (MeOAc, 98%) were purchased from Shanghai Macklin Biochemical Co., LTD. Ethanol (AR), isopropanol (AR), acetone (AR), toluene (AR) were purchased from Sinopharm Chemical Reagent Co., LTD. Cesium carbonate (Cs_2_CO_3_, 99%) and stannous chloride dihydrate (SnCl_2_·2H_2_O, 98%) were purchased from Aladdin Reagent Co., LTD. Lead iodide (PbI_2_, 99.99%) and methylammonium iodide (MAI, 99.5%) from Greatcell Solar Materials Pty., Ltd. *N*,*N*-dimethylformamide (DMF, 99.8%) and dimethyl sulfoxide (DMSO, 99.7%) were purchased from Sigma-Aldrich (Shanghai) Trading Co., LTD. Fluorine-doped tin oxide (FTO) was purchased from YouXuan Technology Co., Ltd. Carbon electrode paste purchased from Shanghai MaterWin New Materials Co., LTD.

### Synthesis and purification of CsPbI_3_ QDs

Cs_2_CO_3_ (0.16 g), ODE (6 mL) and OA (0.5 mL) were added to a 50 mL three-neck flask, where the mixture was stirred at 120 °C for 1 h under nitrogen-filled condition. After Cs_2_CO_3_ was completely dissolved and the mixed solution turned transparent and clear, indicating that Cs_2_CO_3_ reacted with OA to form Cs-oleate. PbI_2_(0.3467 g), ODE (20 mL), OA (3 mL), and OAM (2 mL) were added to another three-necked flask and stir at 120 °C for 1 h under nitrogen-filled condition. After the PbI_2_ was completely dissolved, the mixed solution turned transparent and light yellow. Then, the temperature was raised to 170 °C and 2 mL of Cs-oleate solution was immediately injected into the PbI_2_ precursor solution. After the reaction for 5 s, it was cooled to room temperature in ice water to obtain the original solution of CsPbI_3_ QDs. Next, CsPbI_3_ QDs were purified. 24 mL MeOAc was added to the original QDs solution, centrifuged at 8000 rpm for 5 min. and the supernatant was removed. Then, the precipitate was dispersed in toluene and centrifuged at 4000 rpm for 5 min. Finally, the supernatant of the QDs was collected.

### Device fabrication

**Fig. 1:**
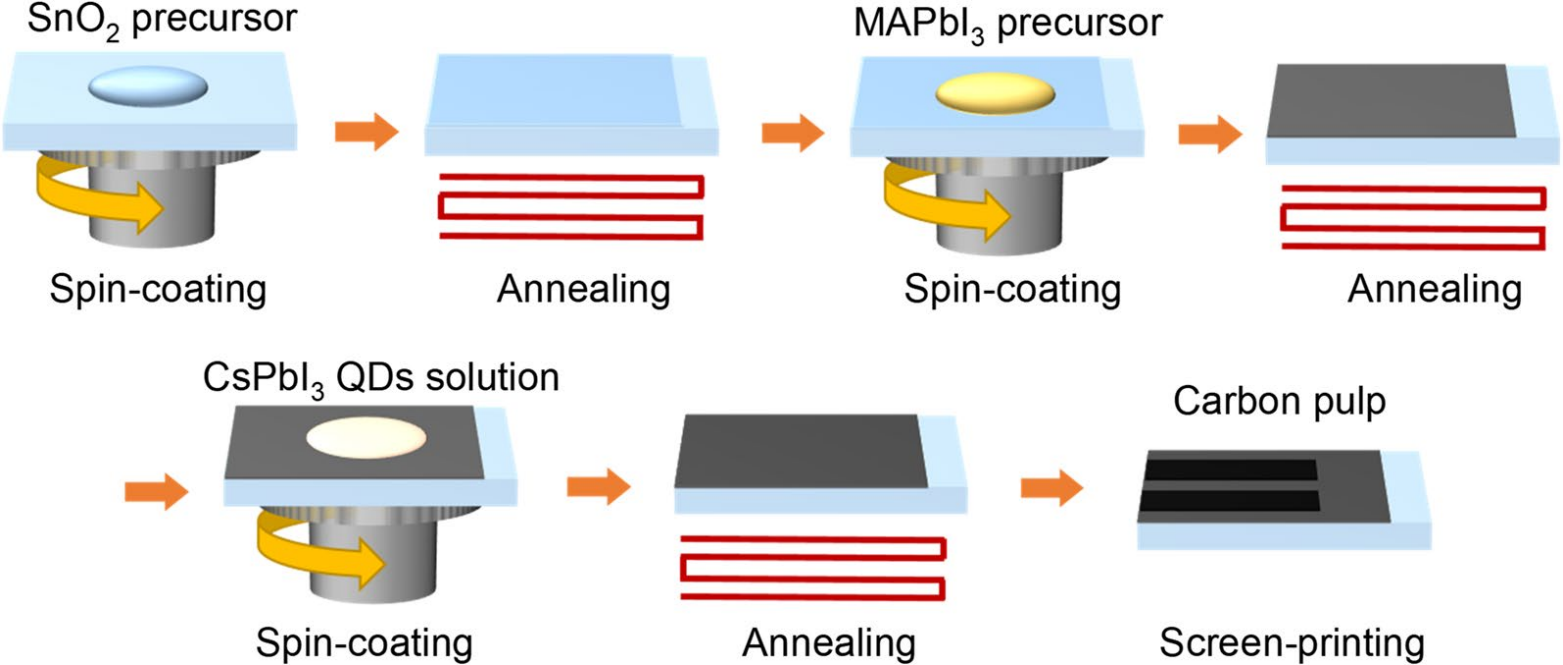
Schematic diagram of the PD fabrication process

Cleaning the FTO conductive glass with a dust-free cloth is followed by ultrasonic cleaning with deionized water, acetone, isopropanol alcohol and ethanol for 30 min each and then blow dry for later use. SnCl_2_·2H_2_O was dissolved in ethanol to produce a 0.1 mol/mL solution, and the SnO_2_ precursor solution was prepared by stirring for 24 h. The perovskite precursor solution was prepared in the glove box. 0.346 g PbI_2_, 0.12 g MAI, 0.45 g DMSO and 0.06 g DMF were added to the brown vial and stirred for 4 h. The FTO conductive glass was further cleaned under the UV-ozone cleaner for 30 min. The PDs device was fabricated as shown in Fig. 1. The SnO_2_ electron transport layer was formed by spinning the SnO_2_ precursor solution onto the FTO at 4000 rpm for 20 s and heating it at 100 °C for 6 min to evaporate off the excess ethanol. Finally, the samples were annealed at 180 °C for 30 min. The perovskite photosensitive layers were prepared in a glove box. First, 35 μL perovskite precursor solution was added to SnO_2_ substrate and rotated at 1000 rpm for 10 s, then at 4000 rpm for 30 s. 150 μL toluene was rapidly added to the substrate within 6 s at the beginning of the second rotation step and then, annealed at 100 °C for 10 min. For the device containing QDs interface, after preparing the perovskite photosensitive layer, 35 μL of CsPbI_3_ QDs solution was added to the perovskite photosensitive layer, rotated at 4000 rpm for 40 s and then, annealed at 90 °C for 2 min. Finally, the substrate was placed under the screen-printing plate and a carbon electrode with an area of 0.06 cm^2^ was scraped. Then, the device was dried at 90 °C for 5 min.

### Characterization

The morphology and size of CsPbI_3_ QDs were characterized by transmission electron microscopy (TEM, JEM-1400Plus, JEOL, Japan). X-ray diffraction spectroscopy (XRD, D8 Advance, AXS, Germany) was used to characterize the crystal structures of CsPbI_3_ QDs and perovskite films. The UV–Visible spectrophotometer (UV-2600, Shimadzu, Japan) is used to measure the absorption of CsPbI_3_ QDs. Steady-state photoluminescence spectra (PL, RF-6000, Shimadzu, Japan) were used to characterize the photoluminescence properties of CsPbI_3_ QDs and perovskite films. Band structure of CsPbI_3_ QDs was obtained by X-ray photoelectron spectroscopy (UPS, ESCALAB 250Xi, Thermo Fisher Scientific, USA). The morphology of the perovskite films was characterized by field emission scanning electron microscope (SEM, JSM-7500F, JEOL, Japan). Time-resolved photoluminescence spectroscopy (TRPL, QM-8000, HORIBA, Canada) was used to characterize the carrier lifetime of perovskite films. The surface roughness of perovskite films was characterized by atomic force microscopy (AFM, Nanoscope IV). The dark current curve is characterized by a digital source meter (Keithley, 4200, America) and a light source (CME-OPS1000, China). The Quantum Efficiency Test System (Newport Corporation, US) is used to characterize the quantum efficiency (EQE) of PDs. Electrochemical Workstation (Zahner Company, Kronach, Germany) measures electrochemical impedance spectroscopy (EIS) of PDs in the frequency range of 1 Hz to 2 MHz in dark conditions with a bias of 0.7 V.

## Results and discussion

**Fig. 2:**
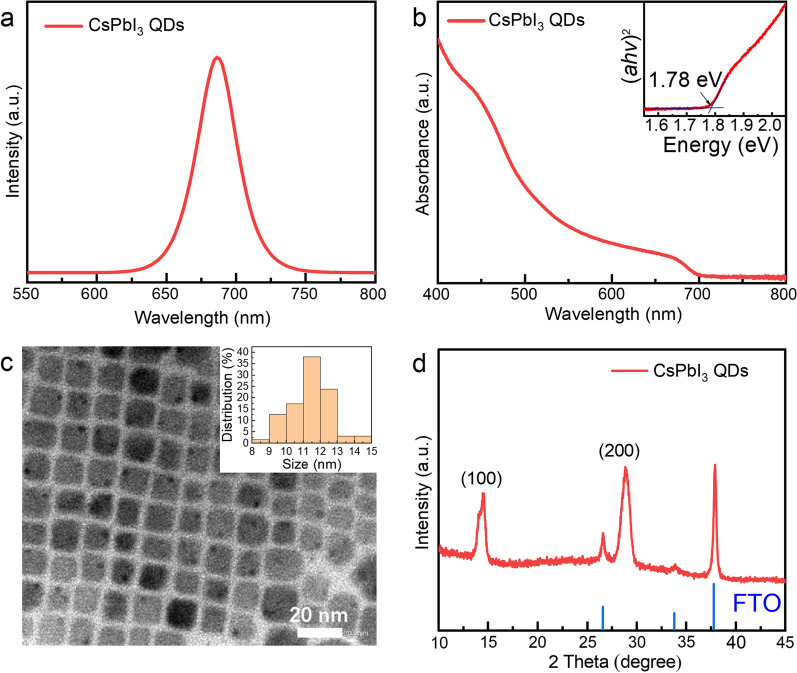
**a** PL spectra of CsPbI_3_ QDs under 365 nm excitation light. **b** UV–Visible absorption spectra and Tauc plot (insert) of CsPbI_3_ QDs. **c** TEM image of CsPbI_3_ QDs. (The inset is size distribution of CsPbI_3_ QDs). **d** XRD pattern of CsPbI_3_ QDs film

The PL of CsPbI_3_ QDs is shown in Fig. 2a, and the emission peak is centered at 686 nm with a narrow full width at half maximum of 32 nm. The UV–Visible absorption spectrum of QDs is shown in Fig. 2b, and its optical band gap has been drawn according to the absorption function curve, as depicted in the illustration [27]. The optical band gap of QDs can be estimated at around 1.78 eV by the Tauc plot, which is similar to previous studies. Figure 2c shows the TEM image of CsPbI_3_ QDs, whose morphologies are cubic. Measured statistics indicate that the average size of QDs is 11.4 nm, which is smaller than the exciton Bohr radius (12 nm) [28]. Figure 2d shows the XRD characterization of the film after spin coating on FTO glass substrate. The diffraction peaks in the XRD pattern correspond to the diffraction peaks of FTO (SnO_2_), CsPbI_3_ QDs cubic phase of (100) crystal plane and (200) crystal plane, and there is no diffraction peak corresponding to orthogonal phase [29].

**Fig. 3:**
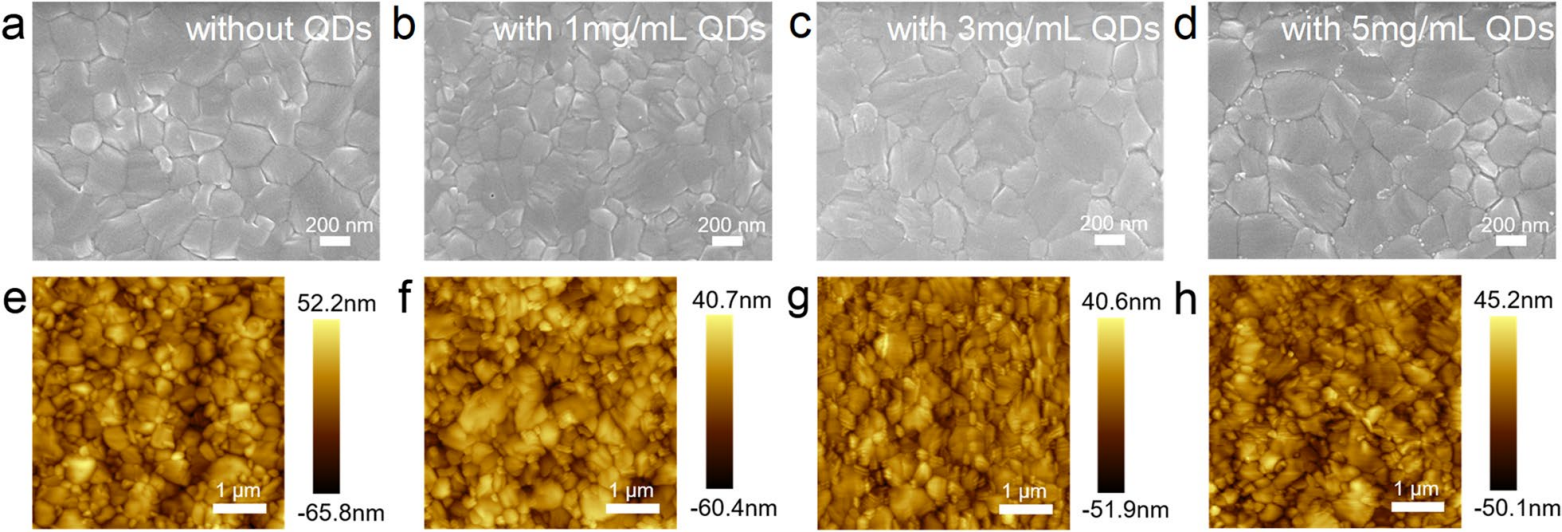
**a-d** SEM images and **e–h** AFM images of control perovskite films, 1 mg/mL, 3 mg/mL and 5 mg/mL QDs passivated perovskite films, respectively

In order to study the influence of QDs concentration on perovskite photosensitive layer morphology, we prepared MAPbI_3_ perovskite films on glass substrate and spin-coated the perovskite surface with QDs at different concentrations. Figure 3a–d shows the SEM images of different perovskite films. Compared to the control perovskite film, due to the low concentration of QDs, it is difficult to clearly observe QDs on its surface. However, as shown in Fig. 3d, the QDs at the perovskite grain boundaries can be clearly observed. Therefore, it is inferred that the QD_S_ are mainly deposited at the grain boundaries of the perovskite films, with a thin layer forming on the surface.

As shown in Fig. 3e–h, the surface roughness of control perovskite films and those passivated by QDs were examined by AFM. In the area of 25 μm^2^, the root-mean-square (RMS) roughness values are approximated to 15.47 nm, 13.62 nm, 10.41 nm and 12.34 nm for the control MAPbI_3_ films and the MAPbI_3_ films passivated by 1 mg/mL, 3 mg/mL and 5 mg/mL QDs layer, respectively. CsPbI_3_ QDs were observed to have a passivation effect on perovskite film surface, which reduces the surface roughness of the film by covering the uneven surface.

**Fig. 4:**
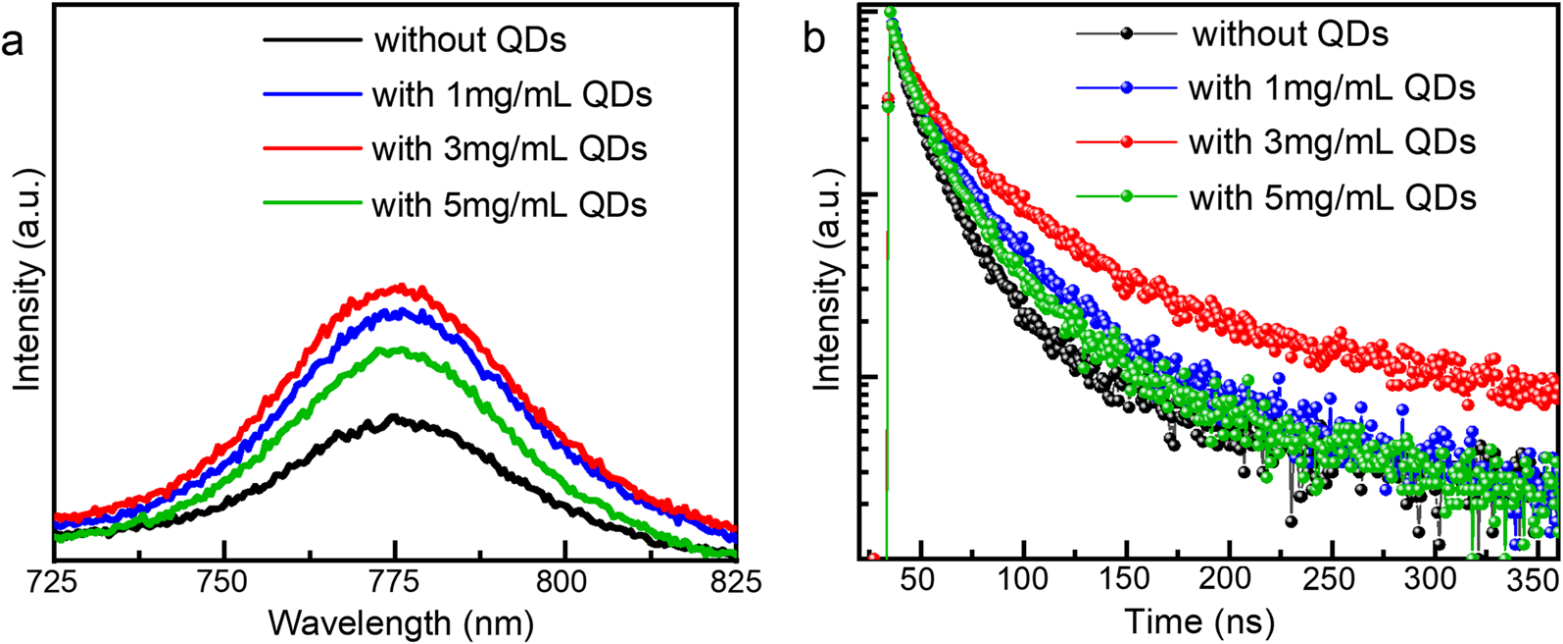
**a** PL spectra and **b** TRPL decay curves of different perovskite films

Figure 4a shows the PL spectrum of different perovskite film on a glass substrate. With the increase in QDs concentration, the PL intensity of the film shows the trend of firstly increasing and followed by decreasing. When the QDs concentration is low, the surface defects of the perovskite film are passivated and reduce, which contributed to the reduced non-radiation recombination and increased PL intensity [30]. On the one hand, the band matching between QDs and perovskite film is primarily responsible for the decrease in PL intensity when the concentration is too intense. At a high concentration of QDs, the carrier extraction of the QDs film is enhanced, which leads to the decrease in the PL intensity [31]. On the other hand, high concentration will increase the thickness of the QDs interface layer, which will increase the roughness of MAPbI_3_ film. The AFM in Fig. 3h proves this result.

In order to further verify the above speculation, we performed TRPL tests on the perovskite films. As shown in Fig. 3b, we fitted the data with a biexponential function. The fitting parameters are shown in Table 1. Obviously, the perovskite film with 3 mg/mL QDs exhibits longer decay (33.969 ns) compared to the perovskite film without QDs (16.126 ns). However, the decay time of perovskite films begins to decline when the concentration of QDs is higher, which is corresponding to the PL spectra. These results show that the films passivated by QDs have lower surface defects and less carrier recombination than the original perovskite films.

**Table 1:** TRPL parameters of different perovskite films

Film structure	τ_ave_(ns)	τ_1_(ns)	Fraction(A_1_)	τ_2_(ns)	Fraction(A_2_)
Without QDs	16.129	4.6594	0.5060	19.1274	0.4715
With 1 mg/mL QDs	23.342	6.8318	0.5173	28.0338	0.4437
With 3 mg/mL QDs	33.969	6.3625	0.4921	38.9774	0.4428
With 5 mg/mL QDs	20.236	6.5495	0.5275	24.7456	0.4238

**Fig. 5:**
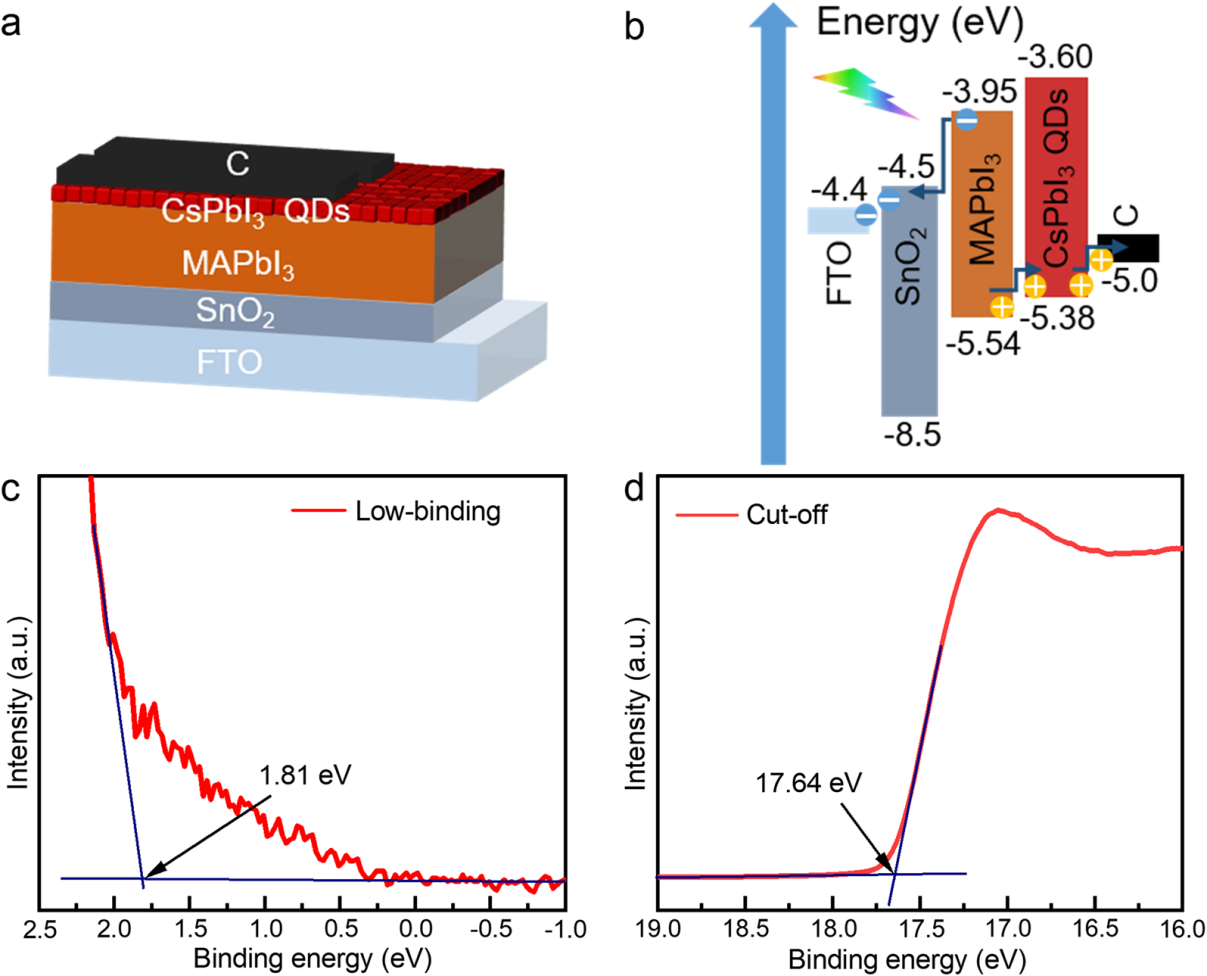
**a** Perovskite PDs device structure diagram, **b** Band energy diagram of the PDs, **c-d** CsPbI_3_ QDs film UPS diagram

The structure of the PDs device prepared in this paper is shown in Fig. 5a, which is composed of FTO substrate/SnO_2_/MAPbI_3_/CsPbI_3_ QDs/Carbon. The control device has no QDs interface layer, and each layer is gradually deposited on FTO by spin-coating. The preparation temperature of the device is not higher than 180 °C. The band energy diagram of the device is shown in Fig. 5b, where the energy level structure of MAPbI_3_ is obtained from previous work. Figure 5c–d shows the UPS spectra of CsPbI_3_ QDs. The valence band (VB) edges and conduction band (CB) edges are calculated by Eqs. (1) and (2) [32].

1 $$E_{{{\text{VB}}}} = - (21.21{\text{eV}} - E_{{{\text{Cut}} - {\text{off}}}} + E_{{{\text{Low}} - {\text{binding}}}} )$$

2 $$E_{{{\text{CB}}}} = E_{g} + E_{{{\text{VB}}}}$$

where *E*_Cut-off_ is the energy of cut-off and *E*_Low-binding_ is the energy of Low-binding. On the basis of the UPS spectrum and Tauc plots of CsPbI_3_ QDs, the edge of the VB and CB of QDs can be determined. As shown in Fig. 4b, the VB edges of the QDs are − 5.38 eV, which is between the VB edges of MAPbI_3_ ( − 5.54 eV) and the VB edges of Carbon ( − 5 eV). The CB edges of the QDs are − 3.6 eV, higher than the CB edges of MAPbI_3_ ( − 3.95 eV). Under light illumination, MAPbI_3_ can absorb light and generate electron-holes, which are extracted by the SnO_2_ film and carbon electrode after separation at low bias. A suitable energy level arrangement of the valence band will enhance carrier extraction. Due to the high conduction band position, the interface of QDs forms an electron barrier layer. This prevents the flow of electrons to the carbon electrode and thus, avoids carrier recombination at the interface [33].

**Fig. 6:**
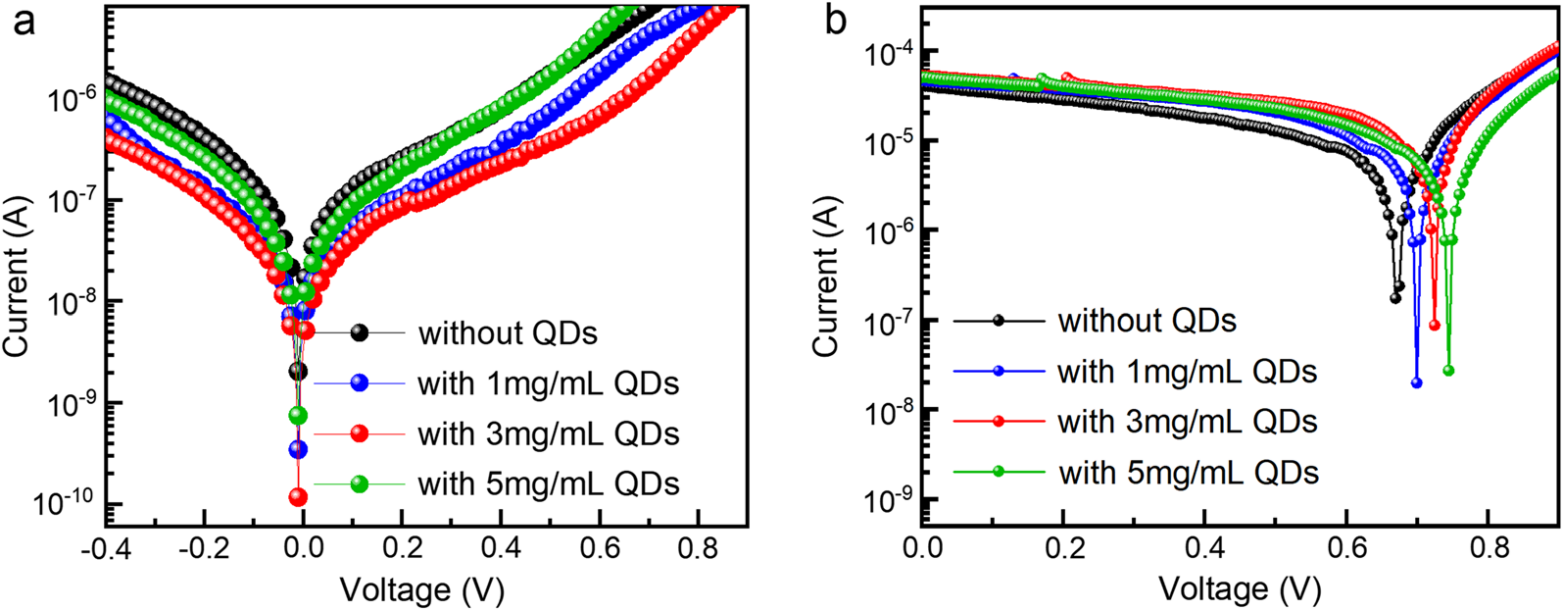
**a** Dark *I-V* curves and **b** Light *I-V* curves of different devices

For further confirmation of the influence of the QDs interface on devices, we measured the light current and dark current of the PDs passivated without and with different concentrations of QDs. Figure 6a shows the dark current–voltage (*I–V*) curves of different PDs. It can be seen that the dark current of PDs without QDs passivation is as high as $2.04 \times 10^{ - 9}$ A, which is mainly due to the surface defects of perovskite photosensitive layer film and the significant carrier recombination caused by the absence of an electron barrier between MAPbI_3_ photosensitive layer and carbon electrode. After QDs passivation, the dark current of the device starts to decrease, and the device with QDs concentration of 3 mg/mL has the lowest dark current of $1.17 \times 10^{ - 10}$ A. The dark current of the device increases when the QDs concentration increased. This is due to the increased surface roughness of the photosensitive layer and the introduction of more defects as a result of the thicker QDs film. The light *I–V* curve that is shown in Fig. 6b verifies this assumption, which is measured under a 500 nm laser with a light intensity of 2.6 mW/cm^2^. The minimum light current corresponds to the open circuit voltage (*V*_oc_) of the device. Compared with control devices, the light current and *V*_oc_ of devices passivated by QDs are improved. The light current of the device with QDs interface is the highest at 3 mg/mL, while at higher concentrations the light current is not significantly better. This also proves that higher concentration of QDs interface will have an adverse effect on carrier extraction. Therefore, subsequent characterization of optimized devices will be based on devices with a 3 mg/mL QDs interface.

**Fig. 7:**
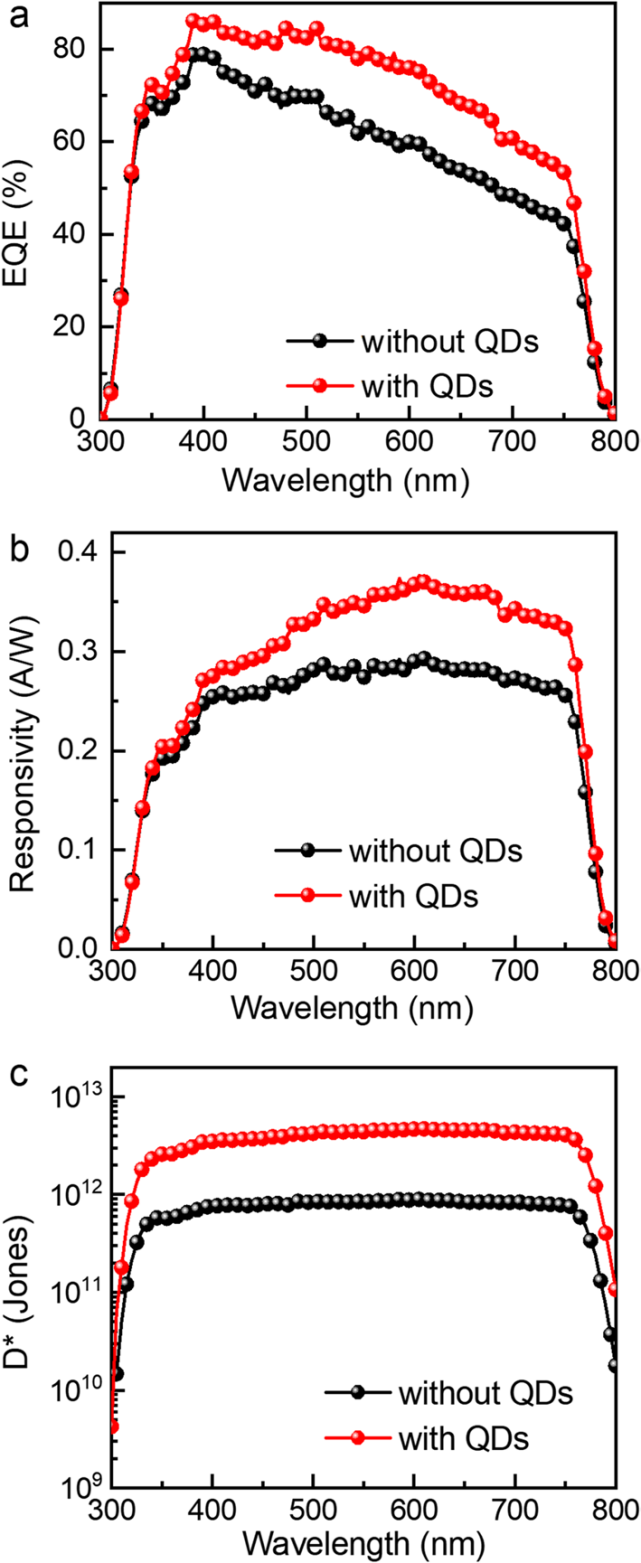
**a** EQE spectra, **b** *R* plots and **c** D* curve of the control device and the device passivated by the QDs interface

The EQE spectra, *R* plots, and D* curves based on control devices and devices passivated by the QDs interface are calculated and provided in Fig. 7a–c. The calculation methods of *R* and D* are given by formula (3) and (4).

3 $$R = \frac{{\lambda \times {\text{EQE}}}}{{{\text{hc}}/e}}$$

4 $$D^{*} = \frac{R\sqrt A }{{\sqrt {2 \times e \times I_{d} } }}$$

where *λ* is the wavelength, *h* is Planck constant, *c* is the speed of light, *e* is the elementary charge, *A* is the device working area and *I*_*d*_ is the dark current [34]. As shown in Fig. 7a–c, it is obvious that the perovskite PDs passivated by the QDs interface have higher EQE, *R*, and D* values. The PDs based on MAPbI_3_ photosensitive layer are all responsive in the visible range. The most obvious increase in EQE and *R* was observed in the range from 500 to 750 nm. Since the long wavelength photons penetrate deeper, the charge carriers excited by these photons are mainly in the photosensitive layer close to the carbon electrode interface [35]. The lower carrier recombination at the interface results in a significant increase in *R* in this wavelength range. The maximum *R* of control device is 0.29 A/W, and the maximum D* is $8.9 \times 10^{11}$ Jones. After passivation by the QDs interface, the maximum *R* is 0.37 A/W and the maximum D* is $4.7 \times 10^{12}$ Jones. The decrease in dark current can significantly improve the photoelectric performance of the PDs, and D* has the greatest effect.

**Fig. 8:**
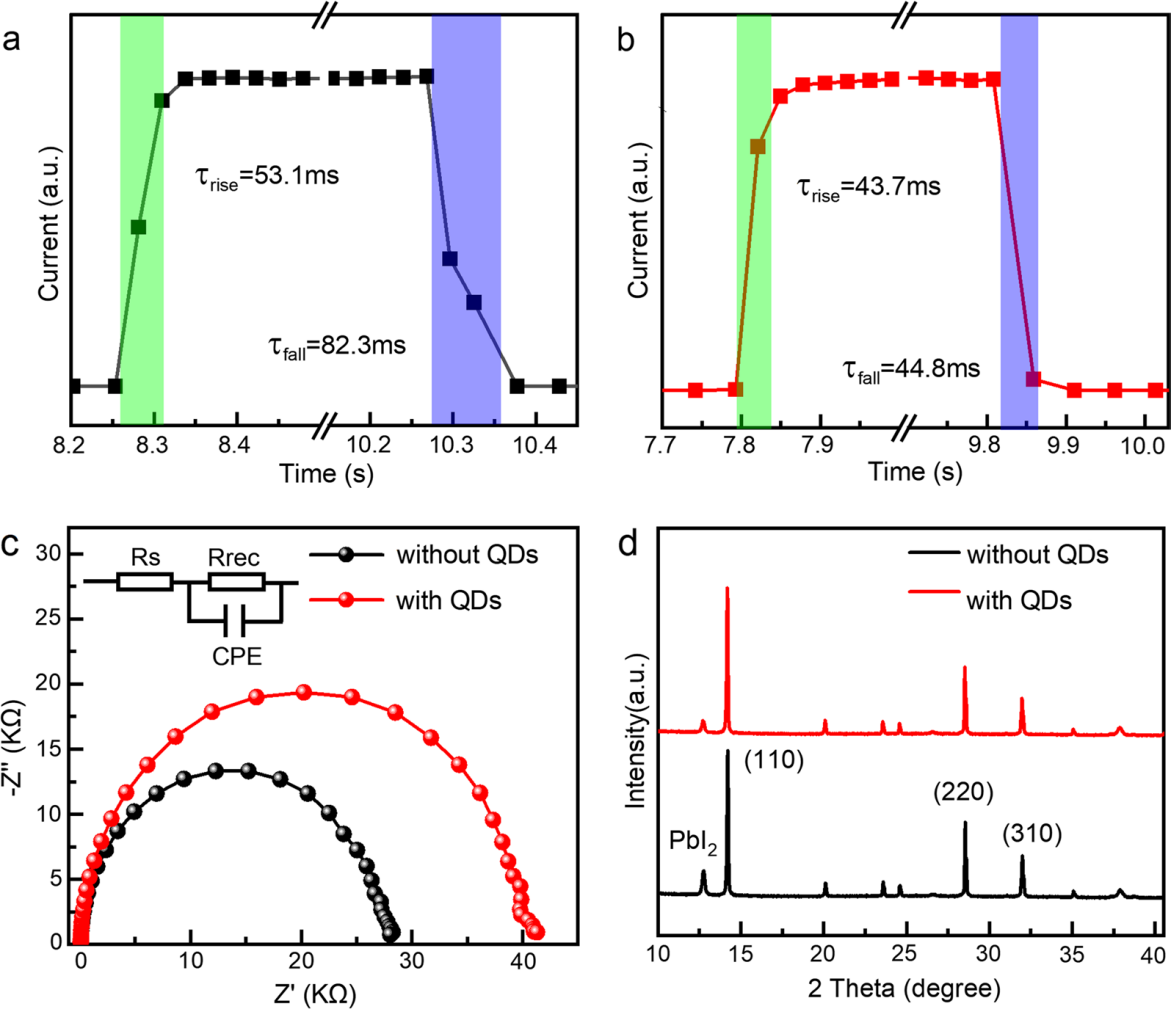
**a-b** Response time diagram of the control device and after passivated by the QDs interface. **c** Nyquist diagram. **d** XRD pattern of the perovskite film one week later

The response time of control device and QDs interface passivated devices are shown in Fig. 8a–b, respectively. Rise time (*τ*_rise_) and fall time (*τ*_fall_) are defined as the duration of the transition between 10 and 90% of the maximum light current and the duration from 90% down to 10% of the maximum light current, respectively [4]. The rise time and fall time of the control device are 53.1 ms and 82.1 ms, respectively. For a device passivated by the QDs interface, the time is 43.7 ms and 44.7 ms, respectively. The faster response time is attributed to fewer interface defects and faster carrier extraction, which is consistent with previous test characterization results. Table 2 summarizes the structure and performance parameters of the device based on MAPbI_3_ perovskite PDs. Performance of the device prepared in this paper is comparable to that of devices with complex structures [4, 36–45].

**Table 2:** Summary of performance parameters and device structure of PDs

Device structure	*R* (A/W)	D* (Jones)	τ_rise_ /τ_fall_	Ref
FTO/TiO_2_/Al_2_O_3_/PCBM/MAPbI_3_/Spiro-OMeTAD/Au/Ag	0.39	10^12^	1.2 μs/3.2 μs	4
FTO/C-TiO_2_/M-TiO_2_ /MAPbI_3_/Spiro-OMeTAD/Au	0.29	3.3 × 10^12^	20 μs/17 μs	36
FTO/Cds /MAPbI_3_/Spiro-OMeTAD/Ag	0.48	2.1 × 10^13^	0.5 ms/2.2 ms	37
ITO/MAPbI_3_ + CuSCN/PCBM/ BCP/Ag	0.37	1.1 × 10^12^	5 μs/5.5 μs	38
FTO/PEDOT:PSS + Ag NPs/MAPbI_3_/Al	0.25	1.5 × 10^11^	110 ms/72 ms	39
FTO/C_60_/MAPbI_3_/CaN/Ln	0.2	7.9 × 10^12^	450 ms/630 ms	40
ITO/ln_2_S_3_/MAPbI_3_/Spiro-OMeTAD/Ag	0.45	1.1 × 10^11^	200 ms/200 ms	41
FTO/ZnO/MAPbI_3_ + CaZnO NRs/MoO_3_/Au	0.34	1.6 × 10^12^	2 ms/2 ms	42
FTO/TiO_2_ + Graphene/MAPbI_3_/ PTAA	0.37	4.5 × 10^11^	5 ms/5 ms	43
ITO/SnO_2_/MAPbI_3_ + GQDs/ PTAA/Au	-	8.3 × 10^10^	220 ms/159 ms	44
ITO/SnO_2_/MAPbI_3_ MCs/C	0.26	7.0 × 10^11^	80 μs/580 μs	45
FTO/SnO_2_/MAPbI_3_/CsPbI_3_ QD_S_/C	0.37	4.7 × 10^12^	43 ms/44 ms	This work

In order to further understand the reasons for the improvement of device performance, EIS tests were carried out on control device and QDs interface passivated devices, respectively. Figure 8c shows the Nyquist plots of the device, which was tested at 0.7 V forward bias and in the dark condition. The equivalent circuit corresponding to the EIS diagram is shown in the inset, and the semicircle in the figure corresponds to the recombination resistance Rrec of the device. Obviously, the Rrec of devices passivated through the QDs interface is larger, which indicates that less charge recombination processes occur within the devices [44]. The interface passivation of QDs reduces the dark current significantly. The improvement of the light current, EQE, *R*, D* and response time is mainly due to the reduction in defects and the band structure matching at the interface.

In addition, to investigate the environmental stability of the device, the perovskite films without and with QDs passivation were stored at room temperature for one week. These films were characterized by XRD. As shown in Fig. 8d, the diffraction peaks of both samples show the crystal structure of perovskite in square phase. However, the diffraction peak of PbI_2_ is observed at 12.72° [46]. When the QDs interface is passivated onto the device, the ratio of the PbI_2_ specific diffraction peak to the (110) crystal plane diffraction peak is lower. This is due to the hydrophobic effect of the oily ligands on the surface of the QDs [47]. Therefore, the perovskite film decomposition produced less PbI_2_ content after one week. It can be seen that devices passivated with QDs interface not only improve photoelectric performance, but also improve the devices’ environmental stability.

## Conclusions

In summary, to solve the problem of large dark current of carbon-based hole-transport-layer-free perovskite PDs, we added CsPbI_3_ QDs interface passivating material between the photosensitive layer and the carbon electrode. The interface passivation of QDs was proved to reduce the surface roughness of photosensitive layer and the defects at the interface. At the same time, the energy level of CsPbI_3_ QDs was well matched with the photosensitive layer and carbon electrode, thus forming a well-performed electron barrier layer. Therefore, the perovskite PDs with interface passivation of CsPbI_3_ QDs have less charge carrier recombination. It was found that the optimized device has a dark current of $1.17 \times 10^{ - 10}$ A, peak *R* of 0.37 A/W and maximum D* of $4.7 \times 10^{12}$ Jones at 605 nm, which is higher than the control device. These results show that introducing QDs on photosensitive layer is beneficial to the reduction in the interface defects and the photoelectric performance of the PDs. Moreover, the environmental stability is also improved. This work promotes the application of CsPbI_3_ QDs combined with carbon-based hole-transport-layer-free of perovskite PDs, which could have great advantages for the development of perovskite PDs.

## Data Availability

The datasets used and/or analyzed during the current study are available from the corresponding author on reasonable request.

## Abbreviations

PDs: Photodetectors
QDs: Quantum dots
PL: Photoluminescence
BCP: Bathocuproine
C_60_: Fullerene
Spiro-OMeTAD: 2,2′,7,7′-Tetrakis(*N*,*N*′-di-p-methoxyphenylamine)-9,9′-spirobifluorene
PTAA: Poly[bis(4-phenyl)(2,4,6-trimethylphenyl) amine]
OA: Oleic acid
OAM: Oleylamine
ODE: 1-Octadecene
MeOAc: Methyl acetate
MAI: Methylammonium iodide
DMF: *N*, *N*-Dimethylformamide
DMSO: Dimethyl sulfoxide
FTO: Fluorine-doped tin oxide
AFM: Atomic force microscopy
XRD: X-ray diffraction spectroscopy
SEM: Scanning electron microscope
TEM: Transmission electron microscopy
TRPL: Time-resolved photoluminescence spectroscopy
EQE: External quantum efficiency
EIS: Electrochemical impedance spectroscopy
VB: Valence band
CB: Conduction band
